# The role of stress factors in severity of *Cytospora plurivora* in greenhouse and field plantings of 13 peach (*Prunus persica*) cultivars

**DOI:** 10.3389/fpls.2023.1228493

**Published:** 2023-08-11

**Authors:** Stephan T. Miller, Sean Wright, Jane E. Stewart

**Affiliations:** Colorado State University, Department of Agricultural Biology, Fort Collins, CO, United States

**Keywords:** *Cytospora*, drought, salinity, canker pathogen, fruit crop, peach

## Abstract

Understanding the host–pathogen–environmental interactions in a pathosystem is essential for management of diseases and diminished crop yields. Abiotic stressors such as cold damage, water deficit, and high pH soils can be major limiting factors to tree fruit production. Along with decreased yields, these abiotic factors can have direct implications for disease severity within orchards. *Cytospora plurivora* is a ubiquitous fungal canker pathogen in western Colorado, USA and is a major focus in integrated pest management strategies. This research evaluated the influence of biotic and abiotic stress factors on peach tree health. Thirteen peach cultivars were placed under abiotic stress and inoculated with *C. plurivora* in greenhouse and field conditions. Under deficit irrigation, *C. plurivora* infections were significantly larger and more severe in both the greenhouse and field trials when compared with those under the full-irrigation controls. In controlled greenhouse conditions, a positive correlation between lesion size and water potential was evident, but no trend of cultivar tolerance was observed. Furthermore, increase in irrigation water pH, through additions of sodium carbonate and bicarbonate, in the greenhouse trials resulted in decreased leaf water potentials and increased pathogen necrotic tissue volumes (mm^3^). In field trials, there was no positive relationship between lesion size and water potential; trees with the most negative water potentials had the smallest lesions sizes that did not correspond to cultivar, suggesting that other abiotic or biotic factors may be shielding water stressed trees from increased pathogen aggression. This research highlights the importance of proper irrigation and soil pH management as tools for the management of Cytospora canker in peach orchards.

## 1 Introduction

Management of abiotic stressors, such as water deficit, high pH, and cold damage, is vital for agricultural crop success. Abiotic stressors are considered the primary limiting factors of agricultural production, affecting 70% of agricultural crops around the world, especially when stressors occur simultaneously (Kaur et al., 2008; Mantri et al., 2012; Khan et al., 2015; Pandey et al., 2017). For Colorado, and many other western regions including Utah, Wyoming, Arizona, California, Nevada, Northern Mexico, and New Mexico, the Colorado River Basin is a major irrigation and water source (Udall and Overpeck, 2017). In the western United States, multi-decade droughts are occurring with little evidence to suggest increases in precipitation in the future (Udall and Overpeck, 2017). Linked to water deficit, high pH and soil salinization are also projected to cause 30%–50% reduction in agricultural land by 2050 (Wang et al., 2003; Khan et al., 2015). Furthermore, cold damage is considered the major limiting factor to deciduous tree fruit production on a global scale; adverse weather conditions, primarily freeze damage occurring in the spring, can damage fruit set, resulting in massive reductions in crop yield (Rodrigo, 2000).

In 2020, peach [*Prunus persica* (L.) Batsch] and nectarine [*Prunus persica* var. *nucipersica* (Suckow) C.K. Schneid] production totals in China dropped by 500,000 metric tons due to an April snow that decimated fruit yields (USDA FAS, 2020). In 2023, low temperatures and snow were reported in April within the peach growing regions of Northern China, damaging peach flower blossoms. However, the losses are expected to be offset by production increases from peach growing areas in Southern China (USDA FAS, 2023). Within the United States, Colorado, Michigan, and New Jersey experienced substantial yield losses due to spring freezes that occurred in 2020 (USDA NASS, 2020). Colorado saw a drastic reduction in fruit yields, with production amounts plummeting from 14,300 tons in 2019 to only 3,000 tons in 2020 (USDA NASS, 2020). Similarly, in 2022, spring freezes caused California, South Carolina, and Georgia to have estimated yield reductions of 15%, 13%, and 26%, respectively, when compared with that in the 2021 season (USDA NASS, 2022). Diminished crop production from abiotic stressors commonly occurs due to increased host susceptibility to plant pathogens (Pandey et al., 2017).

Along with immediate reduction in fruit totals, cold damage of tree tissues can increase incidence of Cytospora canker, raising concerns for overall tree health and lifespan. Interactions among different abiotic stressors can also occur; increased irrigation has been shown to be linked to increasing cold tolerance and reducing canker severity in peach (Layne and Tan, 1984). In other agricultural systems, compounded abiotic stressors have been shown to increase virulence and occurrence of plant pathogens. Pandey et al. (2017) reported that various pathosystems were compounded with abiotic stressors that increase virulence of plant pathogens. Increased temperatures and/or drought exasperate *Rhizoctonia bataticola* (dry root rot) in *Cicer arietnium* (chickpea), *Erysiphe betae* (powdery mildew) in *Beta vulgaris* (sugar beet), *Macrophomina phaseolina* (charcoal stalk rot) in *Sorghum bicolor* (sweet sorghum), and *Streptomyces scabiee* (common scab) in *Solanum tuberosum* (potato) (Lamey, 1988; Mihail, 1989; Potato Council News, 2011; Sharma and Pande, 2013; Sinha et al., 2019; respectively). Thus, to manage limiting factors in peach production of both abiotic and biotic stress, it is important to explore host–pathogen–environment relationships (Garrett et al., 2006).

Orchard water dynamics, including availability and quality, have direct implications for peach fruit production. Although water deficit irrigation strategies are sometimes employed with minimal differences in quality and/or quantity of harvest yield, it is not well known how sustainable these practices may be in the long term considering potential soil salinization risks, which may increase the risk of disease (Aragüés et al., 2014). Bertrand et al. (1976) correlated water deficits to reduced levels of bark moisture in French prune trees [*Prunus domestica* (L.) ‘French’], which, for one fall season, significantly increased Cytospora canker activity in the following seven months. The authors surmised that drought and mismanaging of irrigation were likely linked with increased levels of Cytospora canker in French prune trees.

Deficit irrigation has also been linked to increased soil pH and salinization (Kaman et al., 2006), which can cause physiological complications for peach fruit production. Peach trees are sensitive to soluble salts with possible yield and growth reductions, depending on the soil and irrigation water salt concentrations (Maas and Hoffman, 1977; Hoffman et al., 1989; Aragüés et al., 2014). Western Colorado, as well as other western states, has characteristically high soil pH, which can lead to tree growth limitations due to nutrient deficiencies in zinc, iron, or manganese (Catlett et al., 2002; Fernández et al., 2008). These deficiencies can lead to decreased leaf chlorophyll concentrations, in fresh and dry weight per leaf area, that can cause a delay in fruit ripeness by 2 weeks (Sanz et al., 1997; Morales et al., 1998). In Colorado, high pH is a major issue in heavily irrigated areas; in fact, almost 404 hectares were estimated to be impacted by excess salts (Cardon, 1998). The Green River Formation, within Delta and Mesa counties where peach production occurs, is considered a world class source of sodium carbonate and sodium bicarbonate (Dyni, 1996). Thus, given the risk of high pH in soils in western Colorado, it is essential for cultural practices to include salt mitigation in irrigation waters and soils to prevent physiological damage to trees.

Across different peach cultivars, evidence of susceptibility to *Cytospora* spp. has been linked to a lack of abiotic stress tolerance. Froelich and Schnabel (2019) reported varying lesion sizes on four tree cultivars on inoculated, excised branches. Although excised branches may be a preliminary indicator of differences among cultivar tissues, water transport in live plants has been considered a factor in differences in tolerance among cultivars (Chang et al., 1989 and Chang et al., 1991). *Cytospora* infections cause dysfunction in the plant vascular system through gum production in transport tissues; xylem blockage and gumming is greatest when trees are inoculated during full foliage and vegetative growth, which further suggests that active xylem translocation is essential for disease development (Hampson and Sinclair, 1973). Chang et al. (1989) and Chang et al. (1991) attributed cultivar differences to water transport maintenance through cankered tissues. These differences included necrotic lesion length with progeny from the Russian germplasm ‘Yennoh’ exhibiting the smallest canker sizes. The results suggest that variation in peach cultivar tolerance to *Cytospora* spp. infections occurs and that some cultivars may be less susceptible or not susceptible to Cytospora canker. Within the edible fig production system (*Ficus carica*), fig canker (*Diaporthe cinerascens*) is a major limiting factor; researchers recently reported a drought-tolerant fig tree cultivar to be non-susceptible to the canker pathogen (Bolboli et al., 2022). Thus, the objectives of this study were to evaluate thirteen peach cultivars, commonly planted in western Colorado, for susceptibility to *C. plurivora* D.P. Lawr., L.A. Holland & Trouillas under drought and high pH conditions in the greenhouse and in field conditions.

## 2 Materials and methods

### 2.1 Greenhouse cultivar susceptibility trials

Thirteen, two-year-old, *Prunus persica* scions grafted on ‘Lovell’ rootstocks were selected from two different breeding programs California and Michigan. Selected scions included Glohaven®, Glowingstar®, Blushingstar®, Starfire®, Newhaven®, Flamin’ Fury PF® 19-007, Flamin’ Fury® PF 23, Flamin’ Fury PF® 24, Redhaven®, O’Henry®, Angelus®, Suncrest®, and Cresthaven®. All selected cultivars, 15 trees per cultivar, received heading cuts and were planted in 56.8-L containers with potting mix (Pro-mix BX, Quakertown, PA, USA). Trees were grown for 2 months (mid-May to mid-July 2018) and watered at full pot capacity in a shade house at Colorado State University, Fort Collins, Colorado. Trees were then transferred to a greenhouse to control for precipitation in water deficit treatments.

Trees were grouped into three treatments: 1. control (100% pot capacity, soil pH 7.0), 2. deficit irrigation (60% pot capacity, soil pH 7.0), and 3. high pH (100% pot capacity, soil pH 9.0). A total of 65 trees per treatment were completely randomized, consisting of 13 cultivars and five tree repetitions per cultivar. To determine water pot capacity, all tree pots were fully saturated with water and left for approximately 1 h to allow excess water to drain. After draining, trees were weighed with a mail scale (Dymo® 400) to determine the 100% pot capacity weight (1.0 g H_2_O/g soil). At each consecutive watering event, plants in the drought treatment were only given enough water to maintain 60% pot capacity weight (0.6 g H_2_O/g soil), whereas plants in the watered treatment were watered to 100% pot capacity weight.

For the high pH treatment, soil pH was adjusted to a pH 9.0 by adding 0.10 g of sodium carbonate and 1.0 g of sodium bicarbonate per liter of water to irrigation water. Water pH was confirmed with a pH gauge (Ecotestr pH1, Oakton instruments, Vernon Hill, IL) prior to watering once a week for 2 months. For pH analysis, soil was collected 2 months after continual application of pH treatments. Soil was collected from the rhizosphere of each 56.8-L pot, and samples were sieved and weighed (2.0 g of soil in 50-mL conical tubes). Each tube was then prepared with 5mL of distilled water, and soil slurries were mixed for 15 min. Slurries were placed in 50-mL conical tubes, and pH levels were recorded with a pH probe (AB150 Fisherbrand™ Accumet™, Fisher Scientific Inc., Waltham, MA, USA).

### 2.2 Field cultivar susceptibility trials

Twelve of the 13 3-year-old *Prunus persica* scions were transported to Colorado State Universities’ Rogers Mesa Organic Agriculture Research Station (Hotchkiss, Colorado) in the fall of 2018. Mortality of Cresthaven® trees occurred; therefore, this cultivar was removed from the field trial. All trees were planted in three rows [0.0307 hectare per row (Google, n.d.)] in a completely randomized manner to avoid treatment bias from the greenhouse trials and to include all varieties into a single row. Trees were grown for 1 year and 9 months before field trials were conducted from June 2020 to August 2020. Average temperature and precipitation during the three experimental months in 2020 were 22.3°C and 0.25 mm, respectively. The monthly temperature and precipitation averages for June, July, and August of 2020 were 69.5°C (0.017 mm), 72.6°C (0.0094 mm), and 74.3°C (0.0045 mm), respectively. Trees were categorized in two treatments: 1. full irrigation (two tree rows) and 2. deficit irrigation (one tree row). The irrigation schedules were calibrated by row. Each row (treatment) consisted of 12 cultivars with at least five tree repetitions per cultivar per row. Sample size for each treatment group varied depending on the survival of the transplanted trees over the establishment period. For the full irrigation (two rows) and deficit irrigation (one row) treatments, a total of 141 trees survived (98 and 43 trees, respectively). To ensure tree uniformity within cultivars across rows, prior to applying irrigation treatments, trunk diameter was collected from a sub-sample of 90 trees; trees were measured 15 cm above the graft union.

Irrigation amounts were based on the estimated readily available water (RAW) requirement of the trees for the clay loam soil profile, and the watering values were derived from recommendations made by Washington State University (Smith et al., n.d.). The recommended irrigation amount of 215.86 m^3^ was used to reach field capacity and fulfill the estimated RAW of the trees in the full irrigation control treatment. For the deficit irrigation treatment, 129.515 m^3^ of water was applied to repeat the 60% water application conducted in the greenhouse trials. The total size of each row was determined to be 0.0307 hectare using Google maps (Google, n.d.) and each row consisted of 33 sprinkler heads. The volume of water to reach 215.86 and 129.515 m^3^ at an area of 0.0307 hectares per treatment row was calculated. Irrigation occurred once per week throughout the 2020 growing season. Pre-dawn water potential (PWP) measurements were taken to compare plant water status to validate the irrigation treatment differences.

### 2.3 Plant water potentials

For the greenhouse trials, leaf water potential (LWP) measurements were taken on all trees at solar midday 2 months after establishment of stress treatments. Climatic conditions during leaf collection included no precipitation and an average temperature of 28.3°C. Leaves were cut from trees, and pressure readings were taken using a Scholander Pressure Chamber (Model 1000; PMS Instrument Company; Albany, Oregon). A magnifying glass was used to determine when the compressed nitrogen had provided sufficient gas to force the internal leaf water to the cut edge of the leaf. Pressure values are represented as negative megapascals. For the field trials, PWPs were assessed prior to sunrise between 0300HR and 0600HR daily for 7 days to follow the soil water curve for an entire irrigation period. A minimum of 15 trees, representing all cultivars, were evaluated within the full irrigation row to ensure minimal water stress, whereas a minimum of 35 trees, again representing all cultivars, were evaluated in the irrigation deficit row. This sampling method ensured that irrigation treatments were affecting PWP across treatments. PWPs were taken daily from the most hydrated day after watering, to the least hydrated day, 1 day before the next watering period. PWPs were also measured during the weeks of inoculation and branch harvest to assess the water status of the trees during these periods. To ensure a proper insertion into the chamber seal, a razor blade was used to make a clean cut of the leaf base away from the petiole.

### 2.4 Fungal inoculations (greenhouse and field)

Cultures of *Cytospora plurivora* isolate CP5.1, used in previous studies (Miller et al., 2019; Miller et al., 2021), were grown on one-half potato dextrose agar (PDA) for 4 days prior to inoculation. Inoculations were made after 2 months of the irrigation treatments to the planted trees. For the inoculations, the outer bark of 2-year-old branches was removed with a 4-mm corer to wound the tissue and expose the cambium; the woody tissue below the cambium and the pith were not exposed. Hyphal agar disks (4 mm in diameter), taken from 4-day-old cultures, were placed on each branch wound and wrapped with Parafilm (American National Can; Chicago, IL, 60631, USA). Within each treatment (for all trials), there were five tree replications per cultivar; within each single tree replicate, five branches were inoculated. The average lesion volume of the five branches within one tree replicate was calculated and recorded as one observation; therefore, there were five observations per cultivar per treatment in all trials. Branches were harvested 8 days after inoculation for the greenhouse trial and 13 days for the field trial, due to some cankers reaching the length of the branch with *C. plurivora* fruiting bodies being observed. Canker sizes were measured using a digital caliper, and volume of the decayed tissue was calculated on the basis of branch diameter and canker length. Koch’s postulates were satisfied by the plating of symptomatic plant tissue on one-half PDA media and confirming *C. plurivora* through cultivar morphology.

### 2.5 Statistical analysis

RStudio was used for statistical analyses, and packages used included lme4, lmertest, pbkrtest, and emmeans (Halekoh and Højsgaard, 2014; Bates et al., 2015; Lenth, 2016; Kuznetsova et al., 2017; R Core Team, 2019). Summary statistics and graphical depictions were created using the plyr and the ggplot2 package, respectively (Wickham, 2009; Wickham, 2011). In the greenhouse and field trials, separate one-way ANOVA models were built for the multiple response variables of water potential (MPa) and/or soil pH. A two-way ANOVA was also used to evaluate differences in lesion size across treatments (pH, water deficit, and no stress) for the greenhouse and field trials (**Tables 1A, B**, respectively). Treatment and cultivar were used as fixed predictor variables, with an interaction term included between treatment and cultivar. In field trials, a two-way ANOVA was also used to evaluate differences of trunk diameter, prior to irrigation treatments, with cultivar and row location as fixed predictor variables, and with an interaction between cultivar and row location (**Table 1C**). In both the greenhouse and field trials, linear regressions were evaluated in Excel (2020) to determine the relationship between lesion volume and plant water potential values. To correct for assumptions of normality and equal variances, greenhouse trial data were square root–transformed.

**Table 1:** Two-way ANOVA tables: **(A)** Greenhouse trials consist of three treatments: 1. control [100% pot capacity, soil pH 7.0 (n = 59)], 2. deficit irrigation [60% pot capacity, soil pH 7.0 (n = 60)], and 3. high pH [100% pot capacity, soil pH 9.0 (n = 63)]. **(B)** Field trials consist of two treatments: 1. full irrigation [two tree rows (n = 98)] and 2. deficit irrigation [one tree row (n=43)]. **(C)** Field trials consist of a sub-sample of 90 DBH (diameter at breast height) measurements recorded before irrigation treatments were applied.

(A) Greenhouse trials; response = lesion volume.					
Predictors	*df^a^*	Sum of squares	Mean squares	*F^b^*	*p^c^*
Treatment	2	3,801,701	1,900,850	95.98	< 2E-16
Cultivar	12	438,818	36,568	1.85	0.046
Treatment : Cultivar	24	386,250	16,094	0.81	0.716
Residuals	143	2,832,077	19,805		
(B) Field trials; response = lesion volume.					
Predictors	*df* ***^a^***	Sum of squares	Mean squares	*F^b^*	*p^c^*
Treatment	1	1,488,649	1,488,649	26.60	2.808E-7
Cultivar	11	982,902	89,355	1.60	0.11
Treatment : Cultivar	11	697,333	63,394	1.13	0.34
Residuals	117	6,547,423	55,961		
(C) Field trials; response = trunk diameter.					
Predictors	*df* ***^a^***	Sum of squares	Mean squares	*F^b^*	*p^c^*
Cultivar	11	238.62	21.693	1.15	0.34
Row	1	67.83	67.833	3.60	0.063
Cultivar : Row	11	151.20	13.745	0.73	0.710
Residuals	66	1250.32	18.944		

^a^df, degrees of freedom; ^b^F-value; ^c^p-value (probability).

## 3 Results

### 3.1 Greenhouse cultivar susceptibility trials

**Figure 1:**
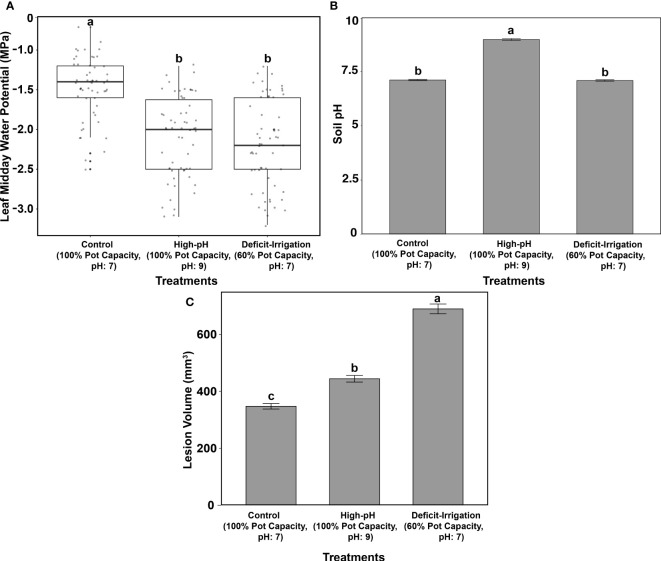
Greenhouse trials included 1) control **(**100% pot capacity, pH 7), 2) high pH (100% pot capacity, pH of 9), and 3) deficit irrigation (60% pot capacity, pH 7 for the 13 cultivars combined). **(A)** Midday leaf water potentials (MPa) across treatments. **(B)** Soil pH across treatments. **(C)** Lesion volume (mm^3^) after *C. plurivora* inoculation. Standard errors are highlighted, and means are labeled with the same letters are not significantly different at *P* = 0.05 according to Tukey’s test.

Differences in LWP (MPa) and soil pH were evaluated across cultivars and treatments to ensure that treatment stress was present (**Figures 1A, B**). Further differences in overall lesion volume were also evaluated (**Figure 1C**). When grouping all cultivars by each of the three treatments, differences in tree LWP were found across the three treatment groups (*P* < 0.0001) (**Figure 1A**). Trees within the high-pH and deficit-irrigation treatments had significantly more negative LWPs when compared with control trees (*P* < 0.0001 and *P* < 0.0001, respectively) (**Figure 1A**) but did not differ between the two stress treatments (*P* = 0.576) (**Figure 1A**). When evaluating soil pH differences between treatments, the high-pH treatment was significantly higher, as expected, than the control and the deficit-irrigation treatments (*P* < 0.0001 and *P* < 0.0001, respectively) (**Figure 1B**), but no difference was observed between the control and irrigation deficit treatments (*P* = 0.884) (**Figure 1B**). Differences in lesion volume did occur (*P* < 2E-16) (**Figure 1C**). The largest lesion volumes were produced in the deficit-irrigation treatment, whereas the smallest lesion volumes were observed in the control treatment (**Figure 1C**). When considering cultivar, the predictor variable in the model yielded a significant value (*P =* 0.046); however, the treatment by cultivar interaction yielded no significance (*P =* 0.716) (**Table 1A**). When further exploring the significance between cultivars, only O’Henry® and FF23® differed significantly from one another (*P =* 0.026), whereas all other comparisons were non-significant (*P* > 0.05).

When trees were subjected to deficit irrigation (60% pot capacity, pH 7) and then inoculated with *C. plurivora*, no differences in lesion volume across cultivars were found (*P* = 0.7819) (**Supplemental Figure 1**). When trees were grown under high-pH conditions (100% pot capacity, pH 9) and compared across cultivars, only Glowingstar® and FF23® showed significant differences between one another, with Glowingstar® developing larger lesions than the FF23® cultivar (*P* = 0.0376) (**Supplemental Figure 2**). When volume sizes on cultivars were compared within the control treatment (100% pot capacity, pH 7), no significant difference among cultivars was observed (*P* = 0.3558) (**Supplemental Figures 1**, **2**).

**Figure 2:**
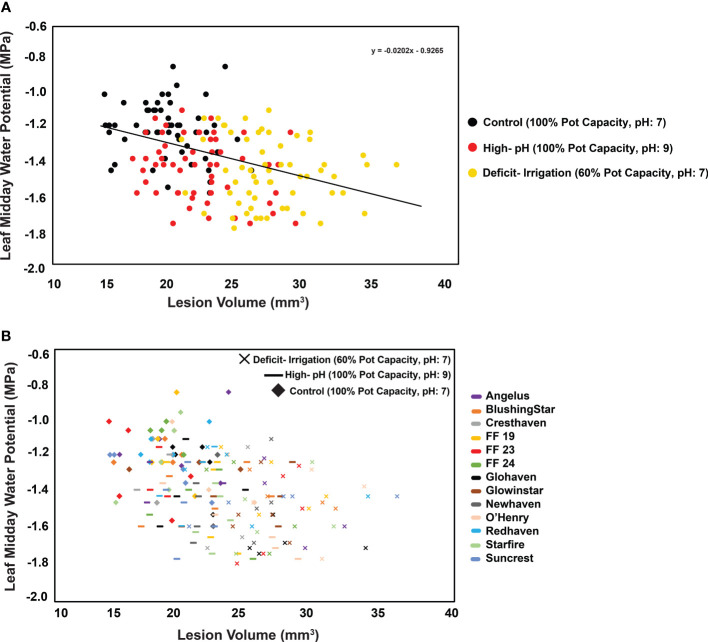
Greenhouse trials: Relationship (R^2^ = 0.1732; *P =* 1.36E-08) between midday leaf water potentials and lesion volume in response to *C. plurivora* inoculations on 13 cultivars of peach trees under different irrigation and pH treatments. **(A)** Three treatments ordered by color: 1) control (black; 100% pot capacity, pH 7), 2) high pH (red; 100% pot capacity, pH 9), and 3) deficit irrigation (yellow; 60% pot capacity, pH 7). **(B)** Same data are shown, but treatment is characterized by shape, and cultivar scion tissue is denoted with colors.

To explore the relationship between the LWP variable and lesion volume variable, a linear regression was evaluated (R^2 = ^0.173; *P =* 1.36E-08) (**Figure 2A**). Trees with the largest lesion volume belonged to the deficit-irrigation treatment (60% pot capacity, pH 7 treatment), whereas trees with the smallest volume and least negative water potentials belonged to the control treatment (100% pot capacity, pH 7) (**Figure 2A**). This regression trend highlighted that trees with more negative LWPs also had larger lesion volumes. However, within treatments (**Figure 2B**), no clustering/grouping by cultivar was observed. Cultivars were highly variable in both LWP and lesion volume values across treatments (**Figure 2B**).

### 3.2 Field susceptibility trials

Prior to applying irrigation treatments, trees showed no statistical difference in trunk diameter when evaluated by cultivar, and there was no row effect by cultivar (*P =* 0.34 and *P =* 0.710, respectively) (**Table 1C**, **Supplemental Figure 3**). To assess differences in tree water status and soil water potential across treatments, PWPs were measured to capture an entire irrigation dry down period (**Supplemental Figure 4**). Trees receiving full irrigation, on average, had significantly higher PWP’s (MPa) at every measurement time point compared with trees subjected to the deficit-irrigation treatment (P < 0.0001) (**Supplemental Figure 4**). After watering, PWP was less negative, but, after 4 days (96 h), trees began to display more negative PWPs. Prior to irrigation on 17 July 2020, trees were most stressed, and the soil water status was lowest. No significant differences were observed between cultivar type within the irrigation-deficit treatment and PWP measurement date (**Supplemental Figure 4**). Dates evaluated included 11 July 2020, 12 July 2020, 13 July 2020, 14 July 2020, 15 July 2020, 16 July 2020, 17 July 2020, 27 July 2020, and 28 July 2020. Comparisons across cultivars within each date yielded the following values (*P =* 0.217, *P =* 0.4304, *P =* 0.9982, *P =* 0.9946, *P =* 0.9131, *P =* 0.7142, *P =* 0.6685, *P =* 0.948, and *P =* 0.1728, respectively).

**Figure 3:**
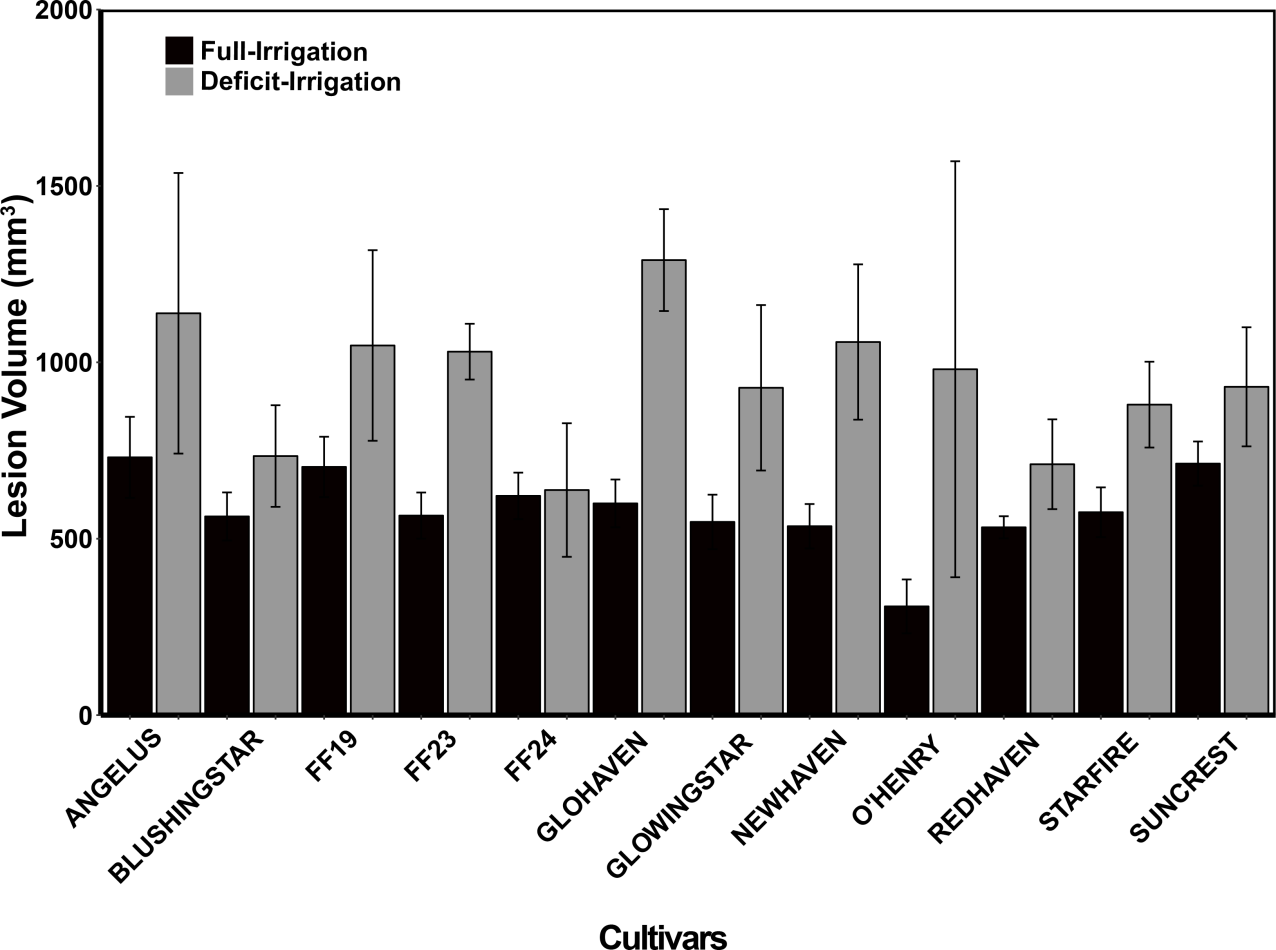
Field trials: Tree necrotic tissue volume (mm^3^) in response to *C. plurivora* inoculations on 12 peach cultivars under two treatment conditions: 1. control (black bars; full irrigation) and 2. deficit Irrigation (gray bars; deficit irrigation). Branch inoculations were made after 3 months of irrigation treatments that were based on the readily available water of the planted area’s soil profile. Standard errors are presented on each bar.

**Figure 4:**
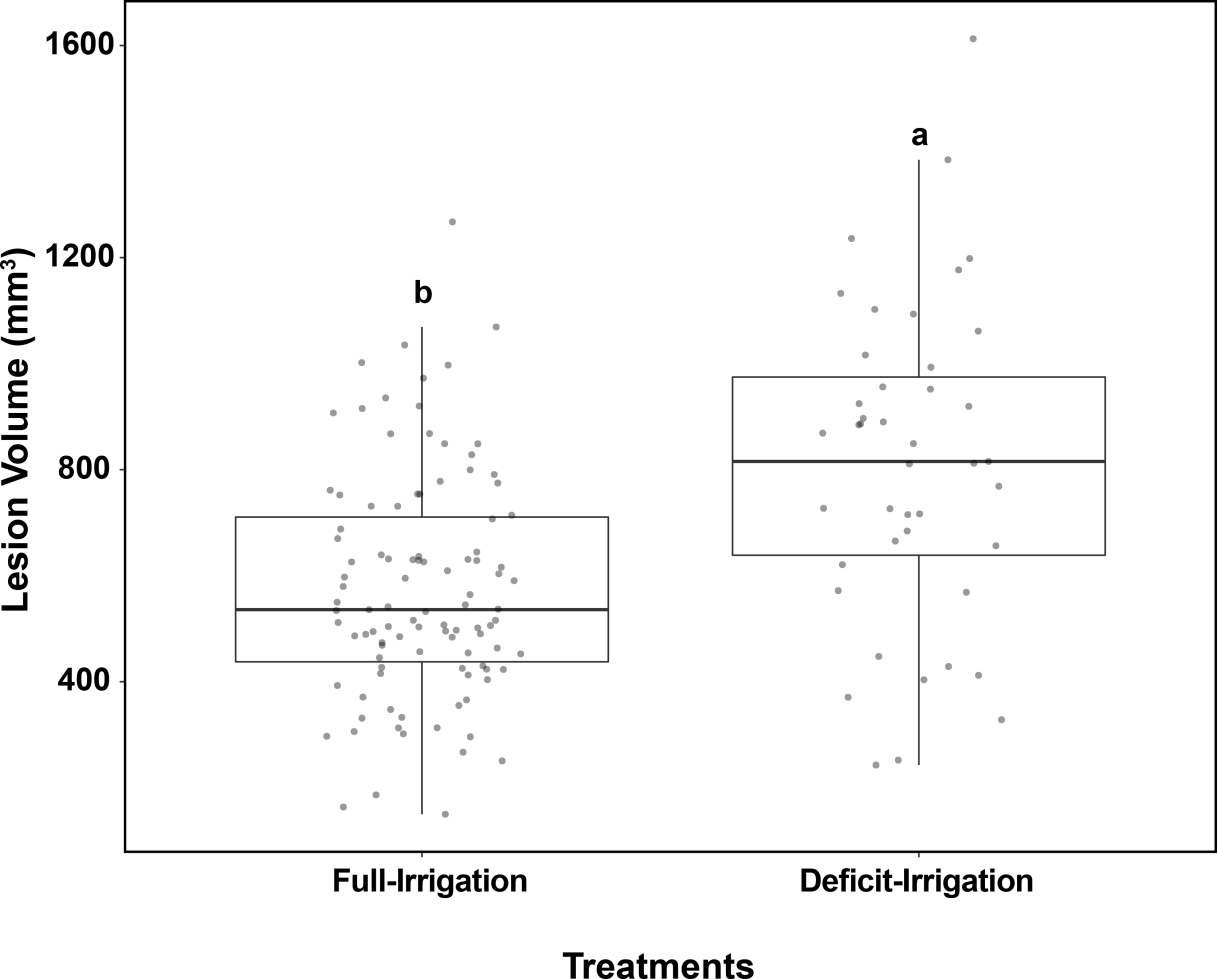
Field trials: Box plots show the effects of full-irrigation/deficit-irrigation treatments based on *C. plurivora* lesion size on peach trees. Response calculated is the necrotic tissue volume (mm^3^). Trees were watered at the given treatments from June 2020 to August 2020. Different letters represent significantly different means (Tukey’s test, *P* = 0.05).

After irrigation treatment applications, trees in the full-irrigation and deficit-irrigation treatments were evaluated for lesion size differences. No lesion volume differences were observed among the 12 cultivars within either the full-irrigation or the deficit-irrigation treatments (*P* = 0.0966 and *P* = 0.5557, respectively) (**Figure 3**). When combining all cultivars, there was a significantly larger lesion volume in the deficit-irrigation treatment with an average lesion size of 808.85 mm^3^, compared to 577.15 mm^3^ in the full-irrigation treatment (*P* = 2.808E-07; **Figure 4**). The treatment by cultivar interaction, in the model with lesion volume as a response, yielded no significance *P =* 0.34 (**Table 1B**).

**Figure 5:**
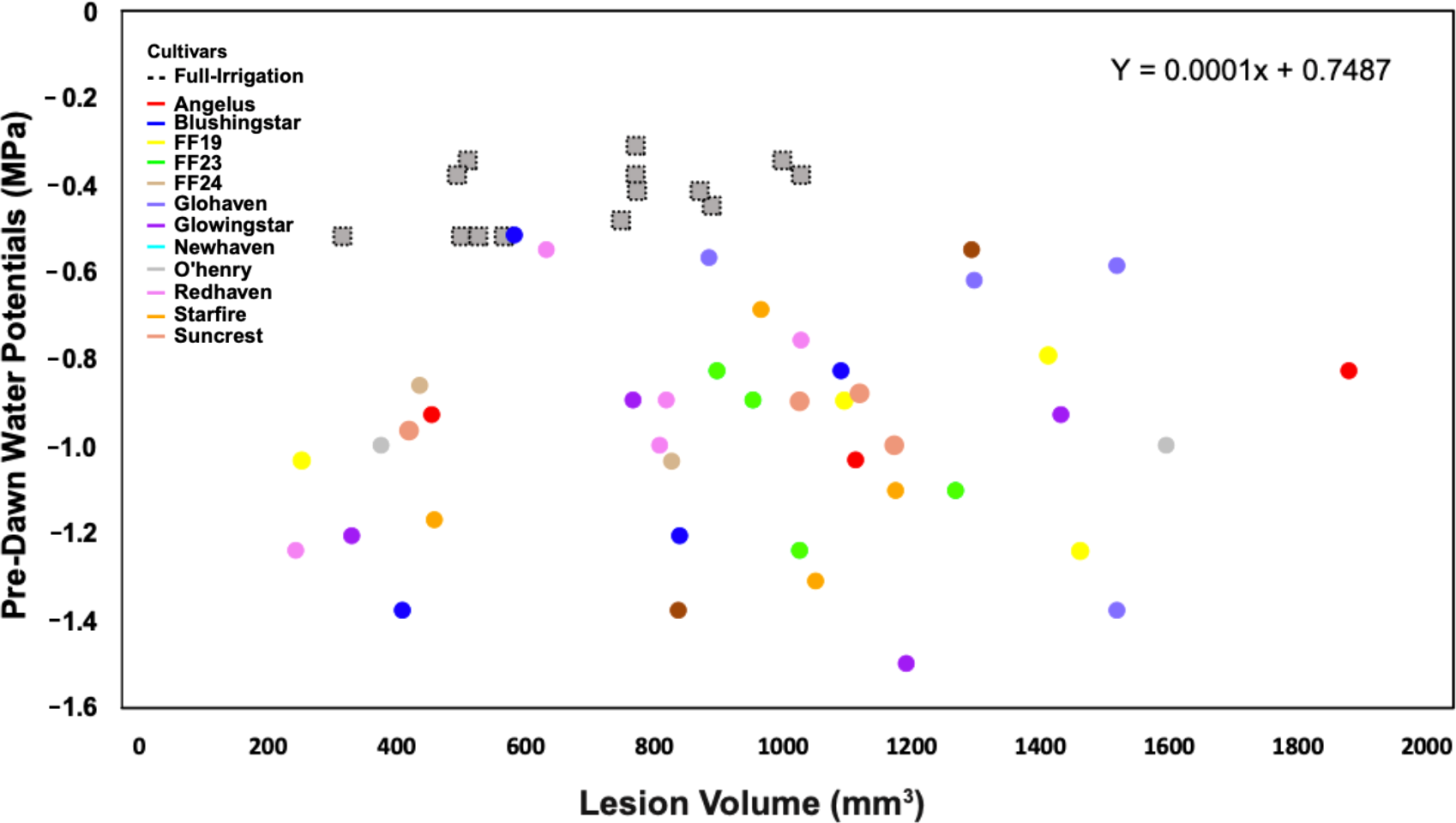
Field trials: No relationship was observed (R^2^ = 0.013; *P =* 0.386) between pre-dawn water potential (PWP) and lesion volume (mm^3^) in response to *C. plurivora* inoculations on 12 cultivars of peach trees under full-irrigation and deficit-irrigation. Treatments are characterized by shape: The full-irrigation treatment is denoted by gray-dotted squares and the deficit-irrigation treatment by circles. Cultivar scion tissue is denoted with colors.

A linear regression explored the relationship between PWP and lesion volume variables, and no correlation was identified (R^2^ = 0.013, *P =* 0.386) (**Figure 5**). Interestingly, some trees in the irrigation deficit treatment, displaying the most negative water potential values, had the smallest lesion sizes (Blushingstar®, Redhaven®, Glowingstar®, and Starfire®, respectively) (**Figure 5**). On average, trees with the largest lesion volumes received the irrigation-deficit treatment. Within treatments, cultivars did not cluster together and were highly variable in both the PWP and lesion volume values (**Figure 5**).

## 4 Discussion

In the greenhouse and field trials, there was a clear overall treatment effect whereby trees under irrigation deficits had significantly larger lesion volumes after inoculation with *Cytospora plurivora*, suggesting that drought stress enhances peach susceptibility. Previous studies have documented similar drought stress effects on plants inoculated with *Cytospora* species (Guyon et al., 1996; Kepley and Jacobi, 2000; McIntyre et al., 1996). Bolboli et al. (2022) reported variation in the susceptibility of different edible fig cultivars (*Ficus carica*) to *Diaporthe cinerascens* with drought-tolerant cultivars being reported as non-susceptible to fig canker. More specifically, in peach systems, researchers have correlated increased *C. leucostoma* growth on trees under water deficit and with reduced levels of bark moisture (Bertrand et al., 1976). Furthermore, increased irrigation rates for peach trees have been reported to decrease Cytospora canker severity and to increase cold tolerance in peach (Layne and Tan, 1984). Presently, there have been no studies evaluating the virulence of the newly described *C. plurivora* species on drought-stressed trees.

Overall, comparisons among tree cultivars inoculated with *C. plurivora* yielded few differences in lesion size in both the greenhouse and field trials. Only O’Henry® differed statistically from FF23® in greenhouse trials, with FF23® having smaller lesion sizes by treatment. Furthermore, no clustering by cultivar occurred in the regression analyses comparing water potentials and lesion sizes for any treatment. As a control, to test differences among scions, all trees were grafted onto ‘Lovell’ rootstock. Our results suggest that susceptibility to *C. plurivora* under water deficit may not be driven by scion tissue; however, there may be an effect of the rootstock influencing the physiological response of the scion. It is well known that the rootstock plays a major role in the physiological signaling within drought-stressed plants; the regulation of stomatal behaviors has been linked to abscisic acid (ABA) production in the roots of drought-stressed plants (Mansfield et al., 1990). In grapevine systems, manipulation of root stock derived ABA, through partial root deficit (PRD) irrigation, can be used to control canopy water use in grapevines plants (Loveys et al., 1998). Rootstock may have a strong influence on scion water potential and/or *Cytospora* susceptibility; nonetheless, despite not observing statistical differences among cultivars, the cultivar Flamin’ Fury® (PF-24C) (FF24) showed similar levels of tolerance under altered water deficit in field trials when compared to full irrigation. FF24 has been bred and marketed as a cold hardy cultivar; thus, it may be that those qualities that contribute to cold hardiness may also play a role in the physiological adjustments within the plant’s self-defense response (Maughan et al., 2016). When analyzing immediate response to shoot wounding, Biggs and Miles (1988) and Wensley (1966) found the rate of callus formation and suberized phellem served as resistance barriers to infections in peaches. Thus, FF24® may form such structures faster than other scion types in response to wounding, freeze wounds, or other wound types exposing underlying tissues.

While the greenhouse trials confirm known literature suggesting a correlation between lesion size and water potential, the field trials did not display the same trend. Interestingly, a selection of water deficit trees displayed the most negative water potentials but had the smallest lesion sizes. There was no linear relationship between water potential and lesion size in field trials despite there being significant differences overall between the lesion sizes due to drought treatment. It is likely that other abiotic and/or biotic variables may be obscuring the relationship. One hypothesis is that there may be an influence of the microbiome present in the field trials. Both fungal and bacterial biocontrol agents have been reported to be effective against Cytospora canker. Yi and Chi (2011) reported significant inhibition of Cytospora canker in poplar stands in China by the cosmopolitan fungi *Trichoderma longibrachiatum.* Hyphal growth of *Cytospora chrysosperma* was significantly inhibited in both the *in vitro* and field trials when exposed to *T. longibrachiatum*. Similarly, researchers reported the ability of *Fusarium* sp. to limit *Cytospora* sp. in poplar trees (Xiang et al., 1991). Zhang et al. (2015) found that the bacterium *Bacillus amyloliquefaciens* significantly inhibited the development of *V. mali* in apple orchards in China. Species of *Trichoderma*, *Fusarium*, and *Bacillus* are ubiquitous in the environment and may have caused antagonism in our study. Future research trials should isolate fungi from tree tissues to evaluate the microbiome present within orchards in western Colorado.

Another hypothesis for the lack of correlation between tree water potential and lesion size in field trials may be the effect of nutrient availability and tree position within an orchard. Nutrient deficiencies may be present unevenly within an orchard row causing physiological advantages or complications despite the watering treatment of a row. Deficiencies in Zn, Fe, or Mn can cause tree growth limitations caused by decreases in leaf chlorophyll concentration, in plant fresh and dry weight per leaf area. This can also result in delays in peach fruit ripeness by 2 weeks (Sanz et al., 1997; Morales et al., 1998; Fernández et al., 2008). Furthermore, differences in microclimates within an orchard may be more or less conducive to *C. plurivora* growth. These differences can be caused by topographical differences within an orchard, location of sprinkler or drip irrigation systems, and/or variation in the surrounding vegetative growth near trees. Such abiotic and biotic variables are potentially influencing field trials and should be investigated in future studies.

In western Colorado, low-quality water sources with high pH are common. Despite maintaining full irrigation schedules in controlled greenhouse conditions, this study documented that irrigation water amended with sodium carbonate and sodium bicarbonate decreases LWP and increases *C. plurivora* lesion sizes. Water stress is a predictor of tree health and measurable through leaf and stem water potential due to anisohydric tendencies of peach trees (Bertrand et al., 1976; Marsal and Girona, 1997; Remorini and Massai, 2003). This study confirms previous literature that has documented that increased salts in soil can have a direct effect on the water potential of anisohydric plants due to changes in root hydraulic resistance altering the flow of water within the plants (Navarro et al., 2007). Other effects of salinity from irrigation water may include mineral deficiency, stomatal blockage, photosynthesis prohibition, cell division prohibition through interaction of salts with plant cellular components, and ion toxicity (Bahmani et al., 2015). When overall treatment effects were compared in the greenhouse trials, trees with increased pH had significantly larger lesion sizes, similar to other studies that have shown that increasing salinity in soils can increase severity of plant diseases (Snapp et al., 1991). Severity of *Phytophthora parasitica* infections to tomato plants was shown to increase with high salinity levels (Snapp et al., 1991). More specifically, in peach systems, studies have shown decreased fruit production totals in response to increased soil pH and salt concentrations (Bernstein, 1980; Hoffman et al., 1989). Correlations between lesion size and water potential were evident in the greenhouse high-pH treatment, suggesting a direct effect from altered pH levels on tree stress. For growers with high pH irrigation waters, sulfur burners may be a viable option for amending irrigation water (Gale et al., 2001) by neutralizing carbonates (CO_3_^2−^) and bicarbonates (HCO_3_^−^) present through the production of sulfur dioxide gas (SO_2_). SO_2_ is dissolved in the irrigation water forming sulfuric acid (H_2_SO_4_), which, in turn, neutralizes carbonates and bicarbonates (Ezlit et al., 2010). Sulfur burners have been shown to be efficient in acidifying stream irrigation water and removing all bicarbonates. Recent studies showed irrigation with sulfur burner–blended water resulted in decreased soil salinity and improved yields and quality of cotton harvests (Ganjegunte et al., 2018). Sulfur burners may be a practical solution for areas with relatively high pH of irrigation water but should be further investigated as there is little research on the actual long term soil effect and physiological effect specific to peach trees.

## 5 Conclusion

It is evident that drought stress enhances peach susceptibility to the recently described *Cytospora* pathogen, *C. plurivora*. Our study documents that, along with drought stress, high pH also increases tree susceptibility to *C. plurivora* and also decreases overall peach tree water potential. Under more controlled greenhouse conditions, there was a positive correlation between lesion size and LWP by treatment, further emphasizing the susceptibility of peach trees under pH and deficit irrigation stress treatments. Thus, cultural practices should ensure that irrigation water quality is acceptable and evenly distributed within fields. Growers should avoid applying irrigation water with high pH and/or concentrations of soluble salts. Furthermore, soils should be tested regularly in areas with high risk of saline soils. Although differences among cultivars were slight, cultivar FF24® showed higher tolerance to *C. plurivora* inoculation in all trials and should be further explored as a hardy scion option for growers. Further exploration into differences among a suite of rootstock may prove useful for identifying sources of tolerance against *C. plurivora*. In addition, future trials should also explore variables that may be obscuring the correlation between water potential and lesion size in field trials. Evaluating the microbiome within peach orchards may yield potential bio-control agents that are serving as antagonists to *C. plurivora*.

## Data availability statement

The raw data supporting the conclusions of this article will be made available by the authors, without undue reservation.

## Author contributions

SM and JS designed studies, JS obtained funding, SM and SW conducted experiments, SM analyzed results and wrote the manuscript, JS and SW edited the manuscript. All authors contributed to the article and approved the submitted version.

## Supplementary material

Supplementary Figure 1 Greenhouse trials: Tree necrotic tissue volume (mm^3^) in response to *C. plurivora* inoculations on 13 difference peach cultivars under two treatment conditions: 1. Control (Black bars; 100% pot capacity) and 2. Deficit-Irrigation (Gray bars; 60% pot capacity). Branch inoculations were made after two months of weekly watering treatments based on pot capacity. Standard errors are presented on each bar.

Supplementary Figure 2 Greenhouse trials: Tree necrotic tissue volume (mm^3^) in response to *C. plurivora* inoculations on 13 difference peach cultivars under two treatment conditions: 1. Control (Black bars; pH of 7) and 2. High-pH (Gray bars; pH of 9). Soil pH adjusted to a pH of 9.0, were made through irrigation water, with sodium carbonate and sodium bicarbonate. Branch inoculations occurred after two months of continuous watering treatments. Standard errors are presented on each bar.

Supplementary Figure 3 Field trials: Tree trunk diameter (mm) of all planted trees grouped by cultivar. Measurements were taken prior to applying deficit-irrigation and full-irrigation treatments. All measurements were taken 15 cm above the graft union. Trees were planted in the fall of 2018 and trunk diameters were measured spring 2020. Means are not significantly different between trees at *P* = 0.05 according to Tukey’s test. Standard errors are presented on each bar.

Supplementary Figure 4 Predawn water potentials (PWP) of peach cultivars in one full-irrigation row and one deficit-irrigation row at 60% RAW according to the soil profile. The average PWP values for the full-irrigation treatment are denoted by a dotted line while the average values for the deficit-irrigation treatment are represented by a solid black line. Colored lines represent PWP values for cultivars within the deficit-irrigation treatment. Bars show standard errors and dotted- vertical lines at 7/10/20 and 7/27/20 represent irrigation dates. Starred full-irrigation averages are significantly different than counterpart deficit-irrigation averages at *P* = 0.05 according to Tukey’s test.
