# Supplementary figures and images for: The role of stress factors in severity of Cytospora plurivora in greenhouse and field plantings of 13 peach (Prunus persica) cultivars
Source: Front Plant Sci. 2023 Aug 11;14:1228493. doi: 10.3389/fpls.2023.1228493 (PMC10452880; doi:10.3389/fpls.2023.1228493)

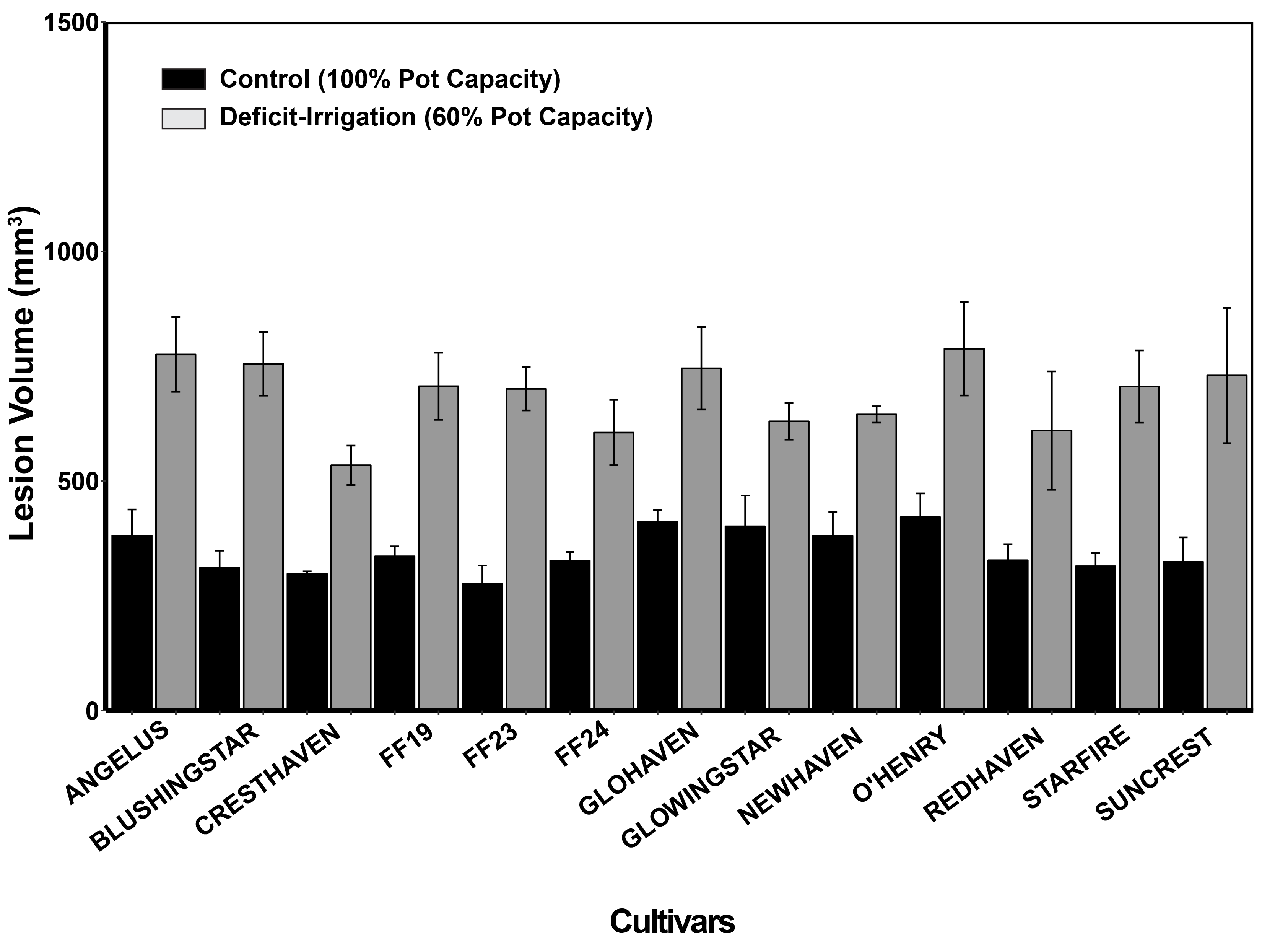

Supplement: Supplementary Figure 1 — Greenhouse trials: Tree necrotic tissue volume (mm3) in response to C. plurivora inoculations on 13 difference peach cultivars under two treatment conditions: 1. Control (Black bars; 100% pot capacity) and 2. Deficit-Irrigation (Gray bars; 60% pot capacity). Branch inoculations were made after two months of weekly watering treatments based on pot capacity. Standard errors are presented on each bar. [file Image_1.tif]

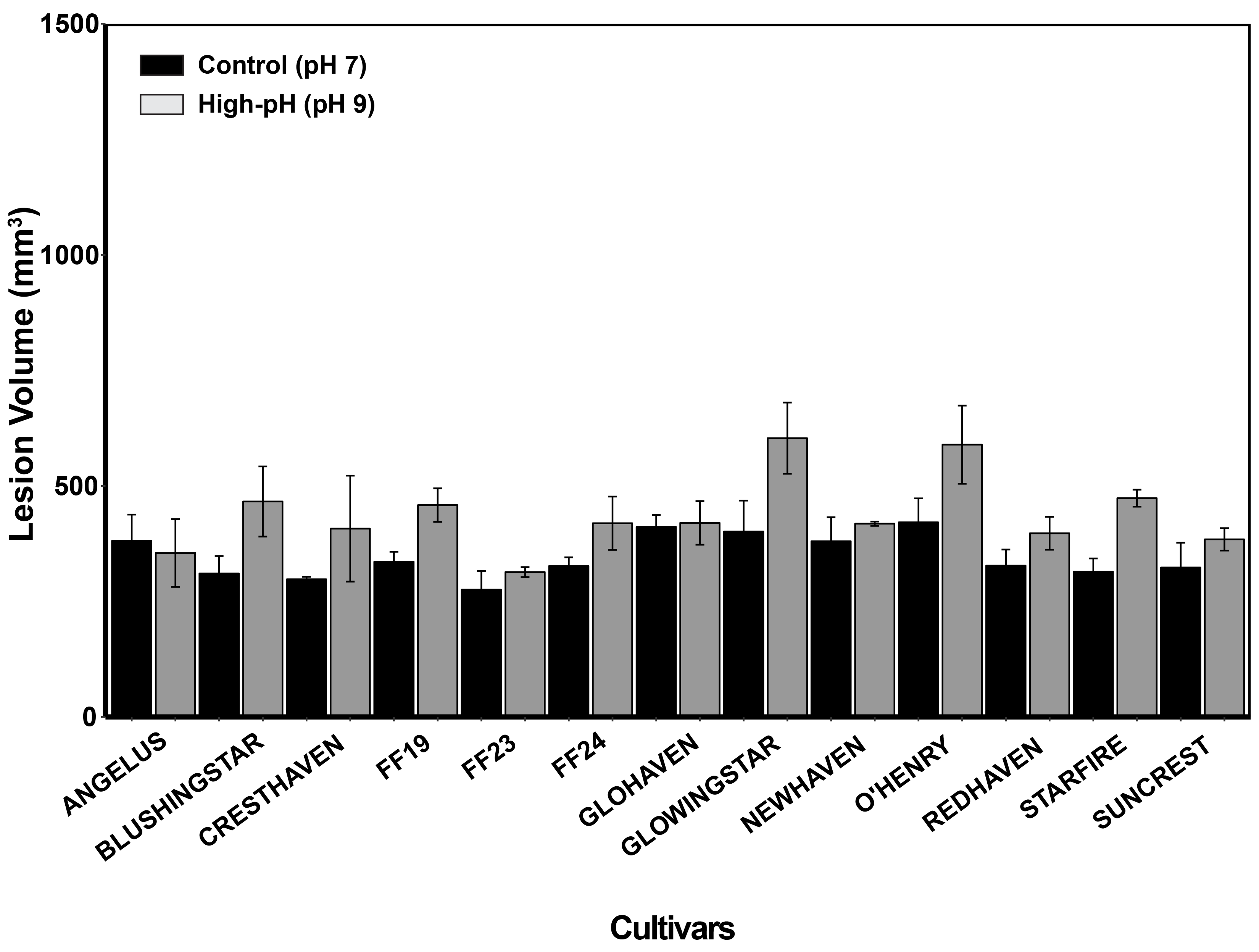

Supplement: Supplementary Figure 2 — Greenhouse trials: Tree necrotic tissue volume (mm3) in response to C. plurivora inoculations on 13 difference peach cultivars under two treatment conditions: 1. Control (Black bars; pH of 7) and 2. High-pH (Gray bars; pH of 9). Soil pH adjusted to a pH of 9.0, were made through irrigation water, with sodium carbonate and sodium bicarbonate. Branch inoculations occurred after two months of continuous watering treatments. Standard errors are presented on each bar. [file Image_2.tif]

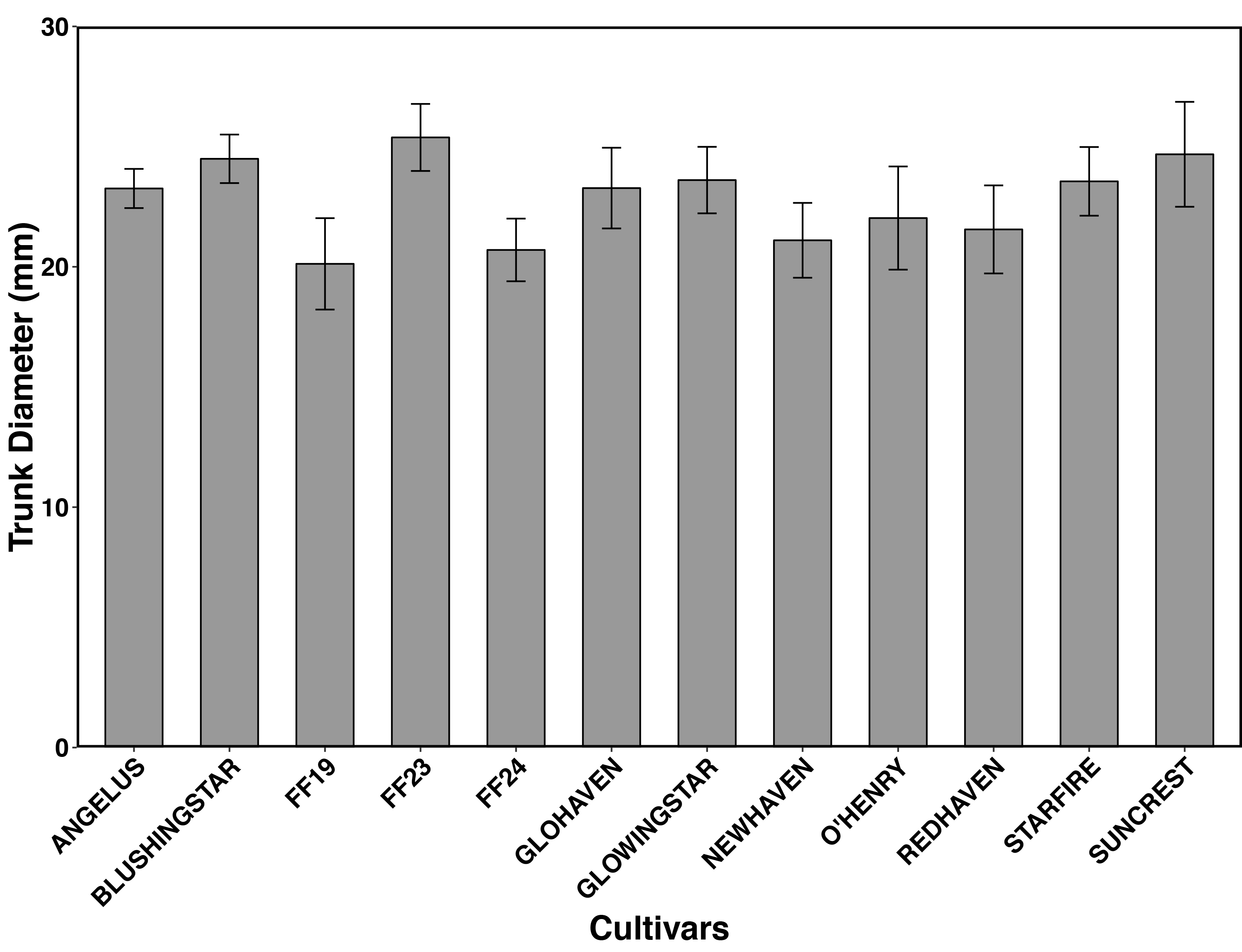

Supplement: Supplementary Figure 3 — Field trials: Tree trunk diameter (mm) of all planted trees grouped by cultivar. Measurements were taken prior to applying deficit-irrigation and full-irrigation treatments. All measurements were taken 15 cm above the graft union. Trees were planted in the fall of 2018 and trunk diameters were measured spring 2020. Means are not significantly different between trees at P = 0.05 according to Tukey’s test. Standard errors are presented on each bar. [file Image_3.tif]

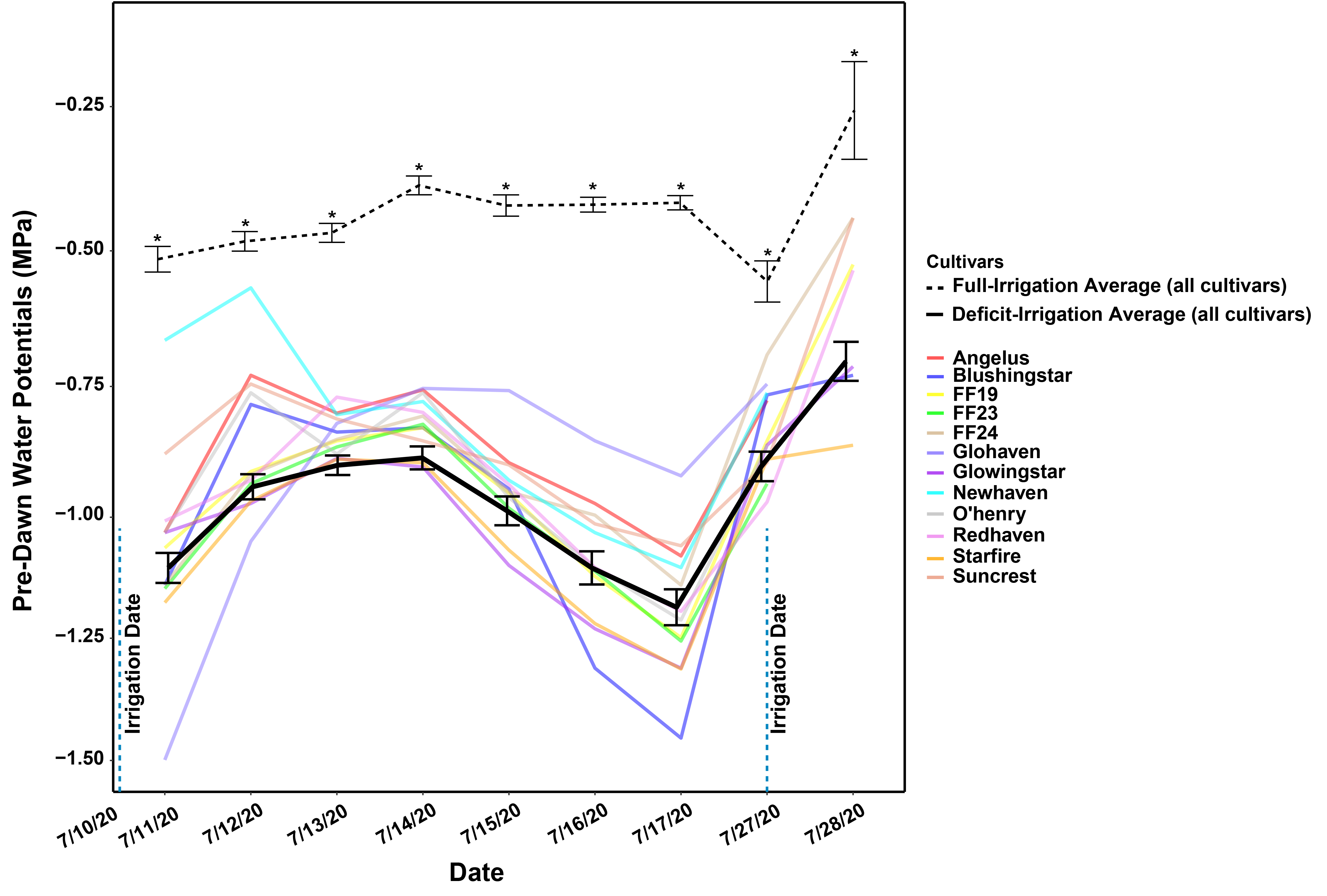

Supplement: Supplementary Figure 4 — Predawn water potentials (PWP) of peach cultivars in one full-irrigation row and one deficit-irrigation row at 60% RAW according to the soil profile. The average PWP values for the full-irrigation treatment are denoted by a dotted line while the average values for the deficit-irrigation treatment are represented by a solid black line. Colored lines represent PWP values for cultivars within the deficit-irrigation treatment. Bars show standard errors and dotted- vertical lines at 7/10/20 and 7/27/20 represent irrigation dates. Starred full-irrigation averages are significantly different than counterpart deficit-irrigation averages at P = 0.05 according to Tukey’s test. [file Image_4.tif]
